# Surgical repair of partial atrioventricular septal defect

**DOI:** 10.12669/pjms.332.12094

**Published:** 2017

**Authors:** Tariq Waqar, Muhammad Usman Riaz, Muhammad Shuaib

**Affiliations:** 1 Tariq Waqar, FCPS, FRCS, Associate Professor of Paediatric Cardiac Surgery, CPE Institute of Cardiology, Multan, Pakistan; 2 Muhammad Usman Riaz, FCPS, Senior Registrar, Paediatric Cardiac Surgery, CPE Institute of Cardiology, Multan, Pakistan; 3 Muhammad Shuaib, FCPS, Registrar, Paediatric Cardiac Surgery, CPE Institute of Cardiology, Multan, Pakistan

**Keywords:** Atrial Septal Defect (ASD), Coronary Sinus (CS), Partial Atrioventricular Septal Defect (AVSD), Pulmonary Hypertension (PH)

## Abstract

**Objective:**

To review the results of surgical correction of partial atrioventricular septal defect and associated cardiac comorbidities.

**Methods:**

Retrospective case analysis of electronic database of department of paediatrics cardiac surgery, CPEIC, Multan was done. Forty consecutive patients operated for partial atrioventricular septal defect repair from September 2011 to October 2016 were included. Mean age was 14.67±7.96 years. 60% (24) patients were male. Regarding echocardiographic findings, pre-operatively 40% (n=16) had mild, 47.5% (19) had moderate and 12.5% (n=5) had severe mitral valve regurgitation. There were 25% (n=10) patients having moderate tricuspid valve regurgitation. Pulmonary hypertension was moderate in 57.5% (n=23) cases and severe in 7.5% (n=3) cases. Among other associated lesions 10% (n=4) patients had secundum ASD, pulmonary artery stenosis was seen in 5% (n=2) patients. Another 5.0% (n=2) patients had bilateral SVCS. While one patient had PDA and one patient had associated common atrium.

**Results:**

Post-operatively there were 19 cases (47.5%) having no mitral valve regurgitation while 18 (45%) patients showed mild and 7.5% (n=3) had moderate mitral valve regurgitation. Only one case had moderate tricuspid valve regurgitation post-operatively, while 22.5% (n=9) cases had mild tricuspid regurgitation. Complete heart block and left sided brain infarct developed in one case. There was no mortality, reoperation, residual atrial shunt or left ventricular outflow tract obstruction.

**Conclusion:**

Repair of partial AV canal carries good overall results with minimal mortality however earlier repair is suggested to reduce post- operative morbidity further.

## INTRODUCTION

Atrioventricular septal defect contributes to 2.9% of congenital heart defects and 60% of these cases are partial atrioventricular septal defects.^1^ Atrioventricular (AV) septal defects are divided into three categories, complete, intermediate and partial. Complete form has both atrial and ventricular septal defects with common AV valve that bridges both sides of heart. Partial AVSD has crescent shaped atrial septal defect above the AV valve and a trileaflet left AV valve leading to its variable regurgitation.^2^ This atrial septal defect is also called primum septal defect. Without significant left AV valve regurgitation, these patients generally present like secundum atrial defects in first decade of life. Marked left AV valve insufficiency and atrial shunt leading to congestive heart failure may need an early corrective surgery.^3^ Otherwise,15% of untreated patients may develop pulmonary vascular disease and atrial fibrillation in their adulthood.^4^,^1^ In 1954, Kirklin and colleagues operated partial AV canal defect first time.^5^ In early era, it was considered that left AV valve is a trifoliate valve different from mitral valve morphologically, so it was not needed to join superior and inferior bridging leaflets. Now most of the surgeons are in opinion that this cleft should be closed to prevent late development of mitral valve regurgitation. Another important consideration is that AV node is displaced posteroinferiorly.^6^ Partial atrioventricular septal defect repairs can result in various possible complications like mitral valve insufficiency, complete atrioventricular node block requiring pacemaker and left ventricular outflow tract obstruction.^7^

Our objective was to review the results of surgical correction of partial atrioventricular septal defect and associated cardiac comorbidities.

## METHODS

It was a retrospective analysis of repair of 40 cases of partial AV canal defects. These patients were operated from September 2011 to October 2016 at Chaudhry Pervaiz Elahi Institute of Cardiology, Multan. Partial Atrioventricular septal defect is a congenital malformation of atrioventricular canal with crescent shaped atrial septal defect above the AV valve but no ventricular septal defect below this valve and have two separate right and left AV valves with cleft in left AV valve. We excluded complete and intermediate forms of atrioventricular canal defects from our study. Also patients of partial AV canal with hypoplastic left ventricle were not included in this study. Moreover we did not encounter any patient with Downs’s syndrome which has high association with complete AV canal.

Approval from ethical and administrative committee of CPE Institute of Cardiology was taken. Twenty four patients were male while 16 were female. Mean age was 14.67±7.96 years. Mean weight was 29.33±15.74 kg. Pulmonary hypertension was moderate in 23 (57.5%) patients while severe in 3 (7.5%). In our study, most of patients had late presentation, so majority of patients were having pulmonary hypertension due to prolonged left to right shunt and progressive left AV valve regurgitation. These patients had oxygen saturation more than 96%, enlarged cardiac shadow and marked pulmonary plethora on chest x-ray and still left to right shunt at atrial level. Therefore, these patients did not undergo cardiac catheterization for assessment of pulmonary hypertension. Regarding echocardiographic findings, pre-operatively 40% (n=16) had mild, 47.5% (19) had moderate and 12.5% (n=5) had severe mitral valve regurgitation. There were 25% (n=10) patients having moderate tricuspid valve regurgitation. Furthermore, 10 patients had other associated lesions along with partial AV canal defects, four (10) patients had ASD secundum, two (5%) had pulmonary artery stenosis and two (5%) had bilateral superior vena cava. While one patient had patent ductus arteriosus (PDA) and one patient had associated common atrium.

We routinely used milrinone (phosphodiesterase inhibitor) and adrenaline as inotropes for termination of bypass. For suspected patients who had preoperative moderate to severe pulmonary hypertension, we continued milrinone in ICU. These patients were kept sedated and paralysed for 12 to 24 hours. Hypoxia, hypercapnia, pain, acidosis and hypothermia were avoided. Our policy was to keep these patients in ICU for 24 hours to observe arrhythmia, bleeding and airway issues. Chest tubes were removed after 24 hours and then transferred them to step down.

### Surgical Technique

After median sternotomy, pericardial patch was harvested and kept wet for later use. Standard CPB was established after cannulating ascending aorta and venae cavae. We cannulated both superior vena cava for bilateral SVCs. PDA was dissected and ligated in one patient. Antegrade cold blood cardioplegia was used for cardiac arrest. Moderate hypothermia was achieved for myocardial protection. After right atriotomy, LV was vented through inter atrial septum. Careful inspection was done to identify if any VSD was also present. We used pledgeted interrupted mattress Prolene 5/0 sutures to sew the pericardial patch with left superior and inferior leaflets to partition between right and left AV valves. Cold saline was injected into left ventricle to assess the mitral valve apparatus. AV valve cleft was obliterated starting at the base of ventricular septum using interrupted simple and mattress sutures to achieve coaptation of left superior and inferior leaflets. After assuring competent mitral valve, continuous Prolene stitches were used to close the remaining inter atrial septum. While proceeding inferiorly, stiches were best placed superficially near the coronary sinus to avoid nodal injury. We incorporated coronary sinus on left atrial side in two patients due to high risk of conduction block.

Cleft was closed partially in two patients to avoid the risk of mitral valve stenosis. We used anuloplasty in six patients by placing commissural stiches at LSL- LLL and at LIL-LLL in five patients and a band was used as a ring in one patient for central leakage. We were not able to repair mitral valve in one patient due to dysplastic left lateral leaflet so mitral valve was replaced with mechanical valve. We encountered accessory mitral valve in one case close to inferior commissure. We left this orifice as such to avoid stenosis. We used large pericardial patch to partition the common atrium. Pulmonary valvotomy was carried in two cases for valve stenosis. Secundum atrial septal defects were closed primarily in three patients while autologous pericardium was used for closure of remaining one. Tricuspid valve repair was done in 10 (25%) patients.

### Statistical Analysis

Following patient related search variables i.e. Age, Gender, weight, Body surface area, disease diagnosis, Surgical procedure performed, total cardiopulmonary bypass time, total cross clamp time, total number of blood transfusion, ICU stay (days), Hospital Stay (Days), in-hospital mortality, operative mortality were searched from Cascade Database. Search results were generated in Microsoft Excel Sheet 2013. Any deficient or dubious data was reconfirmed from hospital paper record. Statistical information was analyzed by using Microsoft Excel 2013 and SPSS Version 16. Mean and standard deviation was utilized to report numeric variable and percentage was utilized to report proportions.

## RESULTS

Mean age was 14.67±7.96 years. 60% (24) patients were male. Mean weight was 29.33±15.74 kg. Mitral and tricuspid valvular regurgitation graded by Doppler color flow with two dimensional echocardiography is shown in Table-I. It also mentioned associated lesions. Operated findings like bypass and aortic cross clamp time are highlighted in Table-II. This table also elaborates postoperative results of valve repair. 19 cases (47.5%) having no mitral valve regurgitation, while 18 (45%) patients showed mild and 3(7.5%) had moderate mitral valve regurgitation. Out of these three having moderate mitral regurgitation, two have preoperative severe left AV valve insufficiency. We repaired tricuspid valve in ten patients with moderate tricuspid regurgitation. Only one case had moderate tricuspid valve regurgitation post operatively, while 9 cases (22.5%) had mild tricuspid regurgitation. Mitral valve was replaced in 19 years old boy after considering it irreparable. Unfortunately that ended up with complete heart block and left sided brain infarct. Permanent pacemaker was placed. He recovered from brain insult and was discharged. There was no residual shunt across atrial septum and patent ductus arteriosus. Echocardiography revealed insignificant gradient across pulmonary valve after pulmonary valvotomy in two patients. We could not see left ventricle outflow tract obstruction in any patient after partial AVSD repair. There was no mortality in our series. During five years follow up we could not observes any deterioration of AV valves, development of left ventricle outflow obstruction or onset of arrhythmias. Three patients with residual moderate mitral valve regurgitation are on close follow up for 2 years for progress of valve insufficiency or left ventricle size and function.

**Table-I:** Echocardiographic Characteristics

*Name of Variable*		*Value*
Age (Y)		14.67±7.96
Male Gender (%)		24 (60.0)
Height (cm)		130.60±29.43
Weight (Kg)		29.33±15.74
BMI (Kg/m^2^)		15.86±2.84
***Echocardiographic Findings***		
Mitral Valve Regurgitation (%)	Absent	0 (0.0)
	Mild	16 (40.0)
	Moderate	19 (47.5)
	Severe	5 (12.5)
Tricuspid Valve Regurgitation (%)	Mild	0 (0.0)
	Moderate	10 (25.0)
	Severe	0 (0.0)
Pulmonary Hypertension (%)	Moderate	23 (57.5)
	Severe	3 (7.5)
***Other Associated Lesions***		
Pulmonary Artery Stenosis (%)		2 (5.0)
ASD Secundum (%)		5 (12.5)
Bilateral SVCs (%)		2 (5.0)
PDA (%)		1 (2.5)
Common Atrium (%)		1 (2.5)

BMI:Body Mass Index, ASD:Atrial Septal Defect, SVC:Superior Vena Cava, PDA:Patent Ductus Arteriosus

**Table-II:** Operative and Post-operative Characteristics.

*Name of Variable*		*Value*
Bypass Time (min)		99.42±30.33
Cross-clamp Time (min)		70.67±30.08
Perioperative Chest Drainage (ml)		381.31±244.20
Ventilation Time (Hours)		5.38±2.59
ICU Stay (Hours)		29.10±13.42
Hospital Stay (Days)		5.81±1.03
***Concomitant Procedures along with Partial AV Canal Repair***	
Pulmonary Valvotomy (%)		2 (5.0)
PDA ligation (%)		1 (2.5)
ASD Secundum Closure (%)		4(10.0)
Post-operative Outcomes		
Mitral Valve Regurgitation (%)	Absent	19 (47.5)
	Mild	18 (45.0)
	Moderate	3 (7.5)
	Severe	0 (0.0)
Tricuspid Valve Regurgitation (%)	Mild	9 (22.5)
	Moderate	1 (2.5)
	Severe	0 (0.0)
Complete Heart Block (%)		1 (2.5)
Left sided Brain Infarct (%)		1 (2.5)

## DISCUSSION

In our study we have found that partial AV canal can present with variable cardiac co morbidities. Most of our patients are late presenters owing to late referrals, with poor prognostic factors like adult age, poor socioeconomical class, with significant number of cases having pre operative moderate to sever regurgitation and pulmonary hypertension. Though reconstructive surgery for AVvalve was possible in most of the patients. With advancements in surgical technique, results for partial AV canal has improved, in current era, hospital mortality is 1% or less.^1^ Weintraub RG, has suggested that post operative moderate regurgitation is significantly increased if there, is pre operative sever regurgitation. Pre operative regurgitation also increases mortality.^7^ Chowdhury UK, et al also suggested that following repair of partial AV canal 10% patients had late sever AV valve regurgitation and most of these patients had preoperatively sever regurgitation.^8^ Manning also suggested that coexisting pulmonary hypertension, complex mitral valve abnormalities raise surgical morbidity.^9^ However early corrective surgery can prevent patient from morbidities like left atrioventricular valvular regurgitation, pulmonary hypertension, arrhythmia and heart failure.^10^ Jeffrey P. Jacobs and Martin J Elliot et al. described that there are 10% chances of left AV valvular insufficiency post operatively, they quoted incidence of permanent heart block less than1% though mean age group in their study for partial AV canal repair was 6.1 years which is earlier than our study population, discharge mortality was 0.3% in their study group while 1% required permanent pacemaker due to heart block, with meanlength of hospital stay 5.2 days.^11^

Jebri et al. also observed in their review of 56 patients having repair for partial atrioventricular septal defect that postoperatively mitral regurgitation was Grade-1 in 28.5% of cases, Grade-2 in 60% of cases while 7.5% cases had grade 3,4 mitral regurgitation.^12^ Moreover, Portman MA, et al. suggested that repair of partial AV septal defect carries low mortality rate however, residual mitral insufficiency is quite common though mild to trivial in most of the cases and often non progressive, they observed significant arrhythmias as a frequent complication often requiring pacemaker implantation in their case series.^13^ Furthermore, Gurbuz AT et al. and co-authors reported that left ventricular outflow tract obstruction after partial AV septal defect repair might be more common than presented in different studies. Therefore they suggested a long term follow up to diagnose such cases as it not only has delayed presentation but also lacks reliable clinical signs.^14^ Though in our study we did not observe any case of left ventricular outflow tract obstruction post operatively.

Our study results are reasonably comparable to these international studies, which is quite encouraging for a paediatric cardiac centre like ours in a developing country though larger and long-term studies should be carried out in future.

## CONCLUSION

Early surgical repair along with long term follow up is highly recommended to allow better preservation of valve structure and to reduce post operative morbidity. Overall Partial AVSD repair carries good results with minimal mortality.

### Author’s Contribution

**TW:** Conceived, designed, prepared this manuscript and is accountable for the originality of the research work

**MUR & MS:** Did data analysis, helped in writing the manuscript.
